# Sleep Disorders and Demand for Medical Services: Evidence from a Population-Based Longitudinal Study

**DOI:** 10.1371/journal.pone.0030085

**Published:** 2012-02-01

**Authors:** Rogerio Santos-Silva, Laura Siqueira Castro, Jose Augusto Taddei, Sergio Tufik, Lia Rita Azeredo Bittencourt

**Affiliations:** 1 Disciplina de Medicina e Biologia do Sono, Departamento de Psicobiologia, Universidade Federal de São Paulo – UNIFESP, São Paulo, Brazil; 2 Disciplina de Nutrição e Metabolismo, Departamento de Pediatria, Universidade Federal de São Paulo – UNIFESP, São Paulo, Brazil; University Medical Center Groningen UMCG, The Netherlands

## Abstract

**Background:**

The aim of this study was to investigate whether insomnia and obstructive sleep apnea (OSA) were predictors of hospitalizations or emergency department visits during two years following the Sao Paulo Epidemiologic Sleep Study (EPISONO) sample.

**Methods and Findings:**

All participants (n = 1,101) who underwent a baseline evaluation between July and December 2007 were contacted in December 2009 and asked to fill out a questionnaire about body weight changes, number of hospitalizations and visits to the emergency department. Participants lost during the follow-up period represented 3.2% (n = 35) and 7 subjects had died. Hospitalizations were reported by 116 volunteers (10.5%) and emergency department visits were reported by 136 participants (12.4%). The average body mass index (BMI) did not vary significantly between the first and the second assessment [26.7(95%CI:26.3–27.1) vs. 26.9(26.5–27.4) kg/m2]. After adjusting for confounders, a multiple logistic regression model revealed that female gender [1.4(1.0–1.9)], age ≥40 years, insomnia diagnosed according to the DSM-IV criteria [1.6(1.0–2.6)], and apnea-hypopnea index ≥15 [1.5(1.0–2.2)] were predictors of hospitalizations and/or demand for emergency services.

**Conclusion:**

Our study of a probabilistic sample of the Sao Paulo inhabitants shows that over a period of two years, insomnia and OSA were both associated with health impairment. Considering the high prevalence and public health burden of sleep disorders, the consequences of untreated disease for both the individual and society are undeniable and should be addressed.

## Introduction

Sleep disorders are increasingly recognized as an important cause of medical morbidity and mortality. Many studies have indicated that the presence of untreated sleep disorders, especially insomnia and obstructive sleep apnea (OSA), is associated with an increased risk of fatal and nonfatal cardiovascular disease [1]–[3], sudden death during sleep [4], death from other causes, stroke [5]–[7], metabolic dysfunction [7], [8], cognitive alterations [9], and impairment in daytime activities [10]. Therefore, the presence of untreated sleep disorders has a substantial economic and social impact but the relationship between sleep complaints and healthcare utilization has not been rigorously evaluated.

The purpose of the present study was to investigate whether insomnia and OSA were predictors of hospitalizations or emergency department visits after a period of two years following up the sample of the Sao Paulo Epidemiologic Sleep Study (EPISONO).

## Methods

The study protocol was approved by the Research Ethics Committee of the Universidade Federal de Sao Paulo (CEP 0593/06), and it was registered at ClinicalTrials.gov (number: NCT00596713). All participants read and signed a written informed consent form.

The baseline evaluation was performed between July and December of 2007 on volunteers enrolled in the EPISONO, a large population-based survey conducted in the city of Sao Paulo, Brazil. The methodological details of this study have been described elsewhere [11]. Briefly, we used a three-stage cluster sampling technique with unequal selection probability to obtain a representative sample of Sao Paulo inhabitants according to gender, age (20 to 80 years), and socio-economic status [12]. The sample included 1,101 individuals, and it was established to allow prevalence estimates with 3% precision [13]. Pregnant and lactating women, people with physical or mental impairments that prevented self-care, and people who worked every night were excluded from the sample. Socioeconomic information, medical history, medication use, and information about sleep habits and complaints were collected in person during home interviews. All participants were invited to undergo an in-lab, full-night polysomnography (PSG); the refusal rate was 5.4%.

Two years after the baseline evaluation (December 2009), all participants were contacted either by e-mail, telephone, or a home visit. Interviews included a questionnaire on changes in body weight, number and causes of hospitalizations and utilization of any emergency services during the follow-up period.

The following predictors were evaluated during the baseline study and were subsequently used to compare individuals who chose to seek medical care: overweight/obesity determined by a body mass index (BMI)>25 kg/m^2^; diabetes that was self-reported in response to a yes/no question; hypertension in individuals who self-reported taking a prescribed anti-hypertensive medication or had high blood pressure (systolic >140 mm/Hg and/or diastolic >90 mm/Hg) measured in the sleep laboratory; symptoms of insomnia in individuals who self-reported difficulties in initiating, maintaining, and/or very early morning awakening at least three times a week; insomnia as defined by the Diagnostic and Statistical Manual of Mental Disorders (IV) in individuals reporting that the previously mentioned symptoms had occurred in the past thirty days and interfered ‘much’ or ‘extremely’ with daily functioning [14]; and OSA, determined by the severity level on the apnea-hypopnea index (AHI) divided in three groups: 1) non OSA determined by AHI<5 plus AHI between 5 and 14.9 without association with symptoms of excessive sleepiness, loud snoring, fatigue, and witnessed breathing pauses during sleep; 2) mild OSA determined by AHI between 5 and 14.9 in association with symptoms of excessive sleepiness, loud snoring, fatigue, and witnessed breathing pauses during sleep; 3) moderate+severe OSA determined by AHI≥15.

The statistical package used in data analysis was SPSS (version 17) for Windows. A general linear model of repeated measures was applied to compare variations in weight and BMI. Complex sample cross-tabulations were applied to determine the weighed prevalence of ‘seeking medical care’ according to predictors in the 1,042 participants who had a PSG recording performed. To generate crude odd ratios, we used multiple, unadjusted logistic models. Because we decided to perform an exploratory analysis, we adopted the backward Wald method to enter variables in the fully adjusted model with all tested predictors.

## Results

Of the 1,101 participants who comprised the total baseline sample, we were able to locate 1,021 by telephone or mail. The remaining 80 participants were visited at their homes and 45 were located. The total number of volunteers lost represented 3.2% of the initial sample.

Seven participants died between December 2007 and December 2009. The causes of death reported by their relatives were heart disease (two), stroke (two), cancer (two) and murder during an assault (one).

We found no significant changes in the variance of the participants' BMI. In 2007, weight and height were measured in the lab, whereas in 2009, weight was self-reported by the participants (26.7 kg/m^2^; 95%CI: 26.3–27.1 vs. 26.9; 26.5–27.4).

During the two-year period following EPISONO sample, a total of 116 individuals (10.5%) were hospitalized, and 136 (12.4%) required health care or first aid for a variety of reasons. The most frequent causes of hospitalization and demand for emergency services were cardiovascular events and/or high blood pressure (27%), infectious processes or pulmonary diseases (14%), psychiatric problems (6%), diabetes (5%), thyroid dysfunction (4%), cancer (3%), and other (41%) including fracture or musculoskeletal problems, kidney and gastric conditions, and surgeries (one of them to treat OSA). The main predictors of these events were female gender, age ≥40 years, insomnia, and OSA (AHI≥15). Before adjustments were made, BMI>25 kg/m^2^, diabetes, and hypertension also presented as potential factors in causing participants to seek medical attention (Table 1). We performed an alternative adjusted model by adding the variables non OSA, mild OSA and moderate+severe OSA (AHI≥15), along with all other variables listed in the Table 1. In this adjusted logistic model (data not included in the Table 1), the variable OSA did not figure as a risk factor for hospitalizations and/or demand for emergency services (non OSA: OR = 1; mild OSA: OR = 0.66 [95%CI: 0.43–1.02], p = 0.06; moderate and severe OSA: OR = 0.61 [95%CI: 0.35–1.03], p = 0.06).

**Table 1:** Weighted frequency (% [95%CI]) of demographic characteristics, medical history, and sleep habits and complaints of the adult population (n = 1,042) of São Paulo as predictors of hospitalizations or emergency department visits after a two-year follow-up period.

	No visits		Visited ED/hospital			Unadjusted Model**		Adjusted Model***	
	N	% (95%CI)	N	% (95%CI)	p*	OR (95%CI)	p	OR (95%CI)	p
Gender									
Men	371	79.0 (73.8–83.3)	92	21.0 (16.7–26.2)	0.187	1		1	
Women	420	74.1 (96.7–78.1)	165	25.9 (21.9–30.3)		1.6 (1.2–2.2)	0.001	1.4 (1.0–1.9)	0.039
Age categories (yrs)									
20–29	204	88.3 (82.3–92.5)	32	11.7 (7.5–17.7)	<0.001	1		1	
30–39	204	83.9 (78.0–88.5)	44	16.1 (11.5–22.0)		1.5 (0.9–2.5)	0.121	1.6 (0.9–2.7)	0.070
40–49	194	77.0 (68.8–83.6)	60	23.0 (16.4–31.2)		2.0 (1.3–3.3)	0.004	1.9 (1.6–3.2)	0.012
50–59	112	67.5 (58.8–75.1)	53	32.5 (24.9–41.2)		3.4 (2.1–5.6)	<0.001	3.1(1.8–5.3)	<0.001
60–69	55	59.7 (45.5–72.5)	38	40.3 (27.5–54,5)		4.8 (2.7–8.4)	<0.001	4.1 (2.2–7.7)	<0.001
70–80	22	36.2 (16.7–61.6)	30	63.8 (38.4–83.3)		9.3 (4.7–18.3)	<0.001	6.6 (3.2–13.7)	<0.001
Socioeconomic Status									
High	228	78.2 (72.3–83.1)	69	21.8 (16.9–27.7)	0.428	1			
Mid	490	76.4 (72.1–80.2)	163	23.6 (19.8–27.9)		1.1 (0.8–1.5)	0.749		
Low	73	69.2 (50.5–83.2)	25	30.8 (16.8–49.5)		1.2 (0.7–2.0)	0.565		
BMI categories									
≤25 kg/m^2^	312	79.8 (74.5–84.2)	83	20.2 (15.8–25.5)	0.05	1			
>25 kg/m^2^	439	73.8(68.0–78.8)	161	26.2 (21.2–32.0)		1.4 (1.0–1.8)	0.046		
Non-diabetes	708	77.1 (73.1–80.6)	44	22.9 (19.4–26.9)	0.058	1			
Diabetes	216	65.0 (50.5.77.1)	28	35.0 (22.9–49..5)		2.1 (1.3–3.4)	0.004		
Non-hypertension	468	80.8 (77.0–84.1)	128	19.2 (15.9–23.0)	<0.001	1			
Hypertension	264	62.4 (53.7–70.3)	116	37.6 (29.7–46.3)		2.4 (1.6–3.1)	<0.001		
Non-insomnia	321	83.5 (78.9–87.2)	68	16.5 (12.8–21.1)	0.01	1		1	
Insomnia complaints	360	72.2 (66.4–78.3)	143	27.2 (21.7–33.6)		1.8 (1.3–2.5)	<0.001	1.4 (1.0–2.0)	0.053
DSM-IV insomnia	110	67.9 (58.9–77.3)	46	32.1 (22.7–43.1)		1.9 (1.3–3.0)	0.002	1.6 (1.0–2.6)	0.036
AHI categories									
<15	646	79.3 (75.9–82.4)	178	20.7 (17.6–24.1)	0.002	1		1	
≥15	106	62.1 (49.2–73.4)	66	37.9 (26.6–50.8)		2.1 (1.5–30.)	<0.001	1.5 (1.0–2.2)	0.048

ED: emergency department; BMI: body mass index; DSM-IV: Diagnostic and Statistical Manual of Mental Disorders; AHI: apnea-hypopnea index.

*Chi-square: test of independence of rows and columns (for complex samples).

**Logistic regression.

***Backward Wald Logistic regression – Of all variables entered at the 1st step, these were the ones that comprised the final model in the 6th step.

## Discussion

We showed that among a probabilistic sample, female gender, age ≥40 years, insomnia, and OSA were significantly associated with an increased number of hospitalizations and/or demand for emergency services during a short EPISONO follow-up period. Our sample included individuals from the general population and represents the inhabitants of the city of São Paulo between 20 and 80 years of age. In contrast to clinical populations, we evaluated individuals who are much less likely to receive a proper diagnosis or treatment of their sleep disorder.

A recent review by Ohayon suggests that the prevalence of insomnia was already high two decades ago, affecting at least one-third of the population who in most cases, did not receive a proper diagnosis or proper treatment for their condition [15]. Studies on the economic impact of insomnia usually discuss direct costs related to resource consumption (i.e., consultations, exams, and products) and indirect costs related to loss (i.e., accidents, absenteeism, and decreased productivity). One recent study showed that insomniacs were 6.7 times more likely to have required medical treatment [8]. Even though severe insomnia is directly associated with the use of healthcare services, worldwide quantification of costs is scarce [16]–[19]. Nonetheless, the costs of untreated insomnia are significantly higher than the direct costs of its treatment [20]. Increasing awareness of the efficacy and availability of insomnia treatment, among both patients and health care providers, can greatly reduce its impact and total costs for society.

The prevalence of OSA syndrome in the EPISONO cohort is one of the highest ever found (32.9%) [21]. It is important to note that in contrast to most studies, we define this syndrome according to the most recent criteria described by the International Classification of Sleep Disorders (ICSD-2) of the American Academy of Sleep Medicine [22]. OSA is a potential predictor of comorbidity and mortality [6], [7], and also has a substantial economic and social impact. Automobile accidents related to OSA in 2000 are estimated to cost $16 billion [23]. Even after controlling for confounders, leaving patients untreated generates costs twice as high providing treatment [24]. In the US, the annual costs of treating the medical consequences of OSA are estimated to be $3.4 billion [25], even though almost 90% of OSA cases are underdiagnosed [26], especially in populations at lower risk for the disease, such as non-obese women [18], as also suggested by our study.

A recent study showed that among US adults who participated in the National Health and Nutrition Examination Survey, those who reported sleep complaints also had increased healthcare utilization. This study also suggested that sleep disorders appear to be highly underdiagnosed [27].

Interesting enough, our study showed that moderate and severe AHI could be a factor in association to other clinical conditions, which together, figured as predictor of poor health outcome, while the AHI between 5 and 15, with or without symptoms, was not found to be a risk factor associated with poor health outcome. In addition, more than ¼ of the causes of hospitalizations and/or demand for emergency services during the short follow-up period were linked to cardiovascular disease, as described in previous studies evaluating OSA and even insomnia [1]–[3].

Sao Paulo no doubt represents the worldwide trend of increasing levels of obesity (22%), OSA, insomnia, hypertension, and diabetes (among other diseases) in all metropolitan areas. It seems logical to think that promoting good sleep would regulate some metabolic and functional processes and might also treat some of the other conditions described herein.

We must put forth an effort to recognize sleep disorders and also to raise awareness of the need to educate both physicians and the population about sleep, the diagnosis of sleep disorders, the benefit of behavioral and psychological treatments and interventions, and alternatives to pharmacology in individuals with chronic insomnia and/or a comorbid psychiatric disorder. Additionally, we must understand the potential therapeutic effects on obesity and adherence to nasal Continuous Positive Airway Pressure or to oral appliance treatments, which can to help us to treat OSA and other breathing-related sleep disorders.
